# Extrachromosomal circular DNA (eccDNA): an emerging star in cancer

**DOI:** 10.1186/s40364-022-00399-9

**Published:** 2022-07-26

**Authors:** Ruomeng Li, Ying Wang, Jing Li, Xikun Zhou

**Affiliations:** 1 State Key Laboratory of Biotherapy and Cancer Center, West China Hospital, Sichuan University and Collaborative Innovation Center for Biotherapy, Chengdu, 610041 China; 2 State Key Laboratory of Oral Diseases, National Clinical Research Center for Oral Diseases, Chinese Academy of Medical Sciences Research Unit of Oral Carcinogenesis and Management, West China Hospital of Stomatology, Sichuan University, Chengdu, Sichuan 610041 PR China

**Keywords:** eccDNA, Cancer, Apoptosis, Proinflammatory response, Cellular senescence

## Abstract

Extrachromosomal circular DNA (eccDNA) is defined as a type of circular DNA that exists widely in nature and is independent of chromosomes. EccDNA has attracted the attention of researchers due to its broad, random distribution, complex biogenesis and tumor-relevant functions. EccDNA can carry complete gene information, especially the oncogenic driver genes that are often carried in tumors, with increased copy number and high transcriptional activity. The high overexpression of oncogenes by eccDNA leads to malignant growth of tumors. Regardless, the exact generation and functional mechanisms of eccDNA in disease progression are not yet clear. There is, however, an emerging body of evidence characterizing that eccDNA can be generated from multiple pathways, including DNA damage repair pathways, breakage-fusion-bridge (BFB) mechanisms, chromothripsis and cell apoptosis, and participates in the regulation of tumor progression with multiplex functions. This up-to-date review summarizes and discusses the origins, biogenesis and functions of eccDNA, including its contribution to the formation of oncogene instability and mutations, the heterogeneity and cellular senescence of tumor cells, and the proinflammatory response of tumors. We highlight the possible cancer-related applications of eccDNA, such as its potential use in the diagnosis, targeted therapy and prognostic assessment of cancer.

## Introduction

Extrachromosomal circular DNA (eccDNA) is well defined as a kind of circulating DNA that occurs widely in nature and is chromosome-independent. EccDNA was first discovered in 1964, and researchers named it double minutes (DMs) [1]. In subsequent studies, the extensive and random distribution, complex biogenesis and tumor-related functions of eccDNA were gradually discovered. EccDNA is a form of gene amplification that can carry complete gene information, including promoters and enhancer elements upstream of the gene, especially oncogenic driver genes that are often carried in tumors, which have an important contribution to the increased expression of oncogenes and can lead to malignant growth of tumors. It has been demonstrated that overexpression of oncogenes by eccDNA results from both its copy number increase and its own high transcriptional activity. We have gradually understood the categories and structures of eccDNA and classified them as small poly-dispersed DNA (spcDNA), telomeric circles (t-circles), microDNA and extrachromosomal DNA (ecDNA) of different sizes [2]. The origin of different kinds of eccDNA varies; for example, the formation of rDNA generates the rDNA circle, while the t-loop is produced at the telomere [3]. However, it is widely accepted that eccDNA is derived from human chromosomes of all known types of genomic structural sequences, especially repetitive sequences, and up to half of the eccDNA is generated from genetic or pseudogenic regions. EccDNA biogenesis, such as the chromothripsis and DNA damage repair pathways, has been discovered in recent studies [4, 5]. Although the exact mechanisms of eccDNA are not yet clear, eccDNA has regained great interest in disease research, including cancer, as a result of technological advances, such as the latest versions of sequencing tools and superresolution microscopes. In early 2022, the latest edition of the hallmarks of cancer was released, providing a clearer direction for tumor research and new ideas about the function of eccDNA [6].

In this review, we focus on the origin of eccDNA, recent research advances in biogenesis, and update the functions of eccDNA from a new perspective, linking it to the latest 14 hallmarks of cancer and strengthening the link between eccDNA and features such as tumor amplification, heterogeneity and aging. We emphasize the potentially useful applications of eccDNA in cancer, for example, its potential use in the diagnosis, targeted therapy and prognostic assessment of cancer patients. Difficulties in monitoring the presence of early cancers, inhibiting drug resistance in cancer development, and predicting cancer prognosis may be addressed by further studies of eccDNA.

### History of eccDNA

**Fig. 1:**
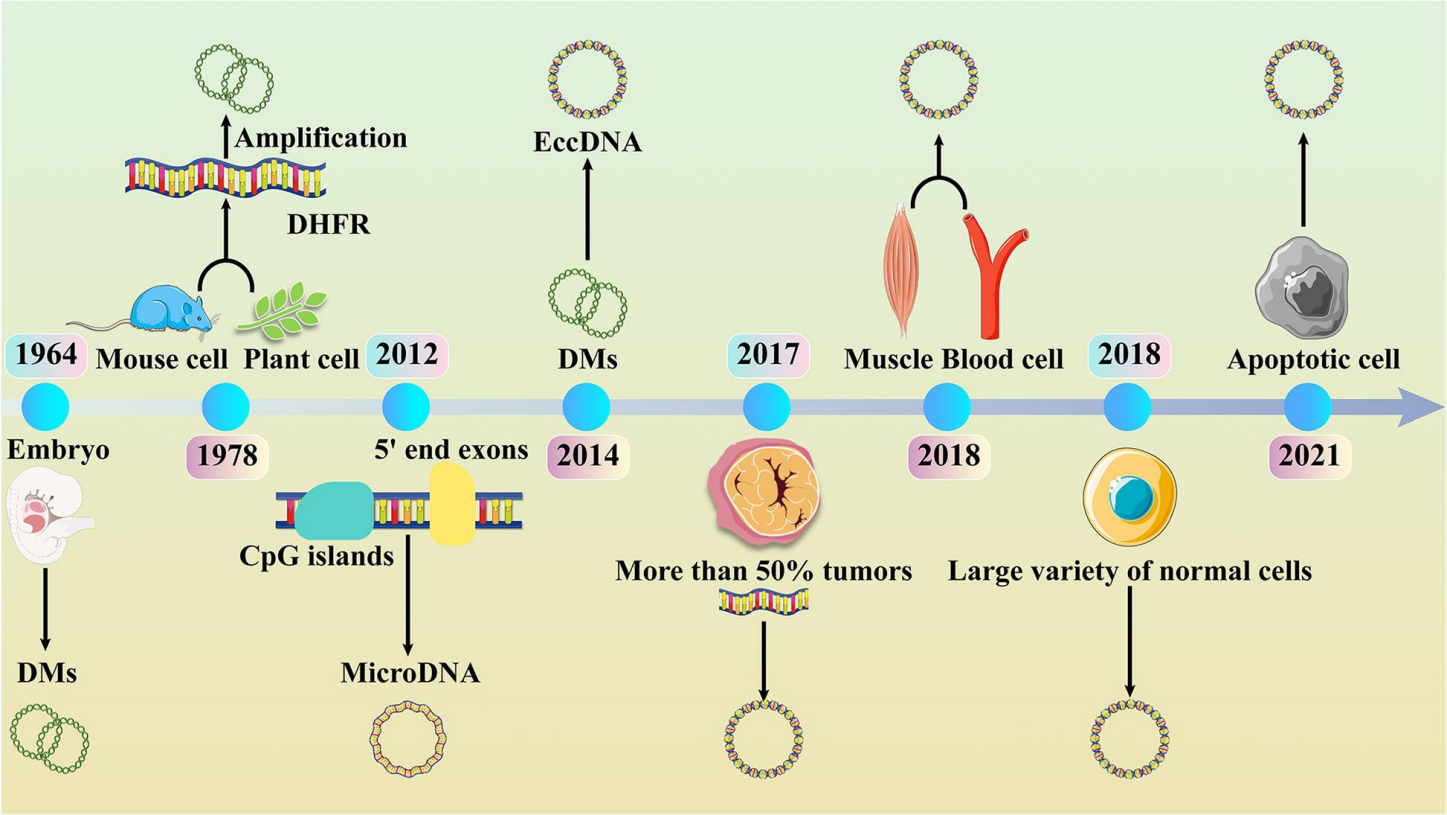
Timeline of the historical milestone for the discovery of eccDNA. Research on eccDNA dates back to 1964, when scientists found double minutes (DMs) in embryos and boar sperm. In 1978, DMs was found to be produced by DHFR gene amplification. MicroDNA was discovered in CpG island and 5’end exons in 2012. Over time, DMs were found in both plants and animals, and their name was changed to eccDNA. At first, eccDNA was thought to be present only in tumor cells, until 2017, it was also found to be present in normal cells as well, such as the muscle and blood cells. Then, in 2021, it was discovered that eccDNA occurs in association with apoptosis, bringing researchers a brand-new way to tackle the issue of oncology

The discovery of eccDNA can be traced back to 1964. EccDNA was first discovered in wheat embryos and boar sperm by Hotta et al. In the same year, Cox et al. found extrachromosomal DNA in childhood malignancies [7]. Because this type of DNA is often found in pairs, it is also called double minutes (DMs). Since then, researchers have found eccDNA in a wide range of species, such as yeast, as well as mammals and plants [8]. The biological role of eccDNA has likewise received a great deal of attention from researchers. Alt et al. in 1978 discovered the presence of eccDNA in mouse cells, which led to dihydrofolate reductase (DHFR) gene amplification and mediated the resistance of mouse cells to methotrexate [9]. As more studies were conducted, it was demonstrated that DMs are capable of carrying oncogenes, including the epidermal growth factor receptor (EGFR) gene [10]. Meanwhile, researchers discovered the circular structure of DMs and found that DMs make up only 30% of eccDNA; this term was gradually replaced by eccDNA [10]. As the study progressed, scientists found that some circular DNAs were abundant in nonrepetitive sequences, 5′ end exons and CpG islands in cells, and these molecules were called microDNA [4]. In 2017, by analyzing the whole genome of 2572 cell lines from 17 tumors, Verhaak et al. discovered that more than half of human tumors contained eccDNA, and these eccDNAs typically carry tumor-driving genes, revealing a possible role for eccDNA in cancer [11]. In 2018, Møller et al. found tens of thousands of eccDNAs in muscle and blood cells of normal humans, most of which carry intact genes or fragments, exemplifying that eccDNA may be prevalent in a variety of cells in normal humans [5]. Since then, eccDNA has gradually become a hot spot of researchers because of its complex biological functions involved in the biological processes of various diseases (Fig. 1).

The biogenesis of eccDNA is still unclear, but studies have been conducted to gradually solve the puzzle. In 2021, Paulsen et al. elaborated more clearly that the generation of eccDNA is related to the damage and repair mechanism of DNA [12]. Wang et al. demonstrated that the production of eccDNA is closely related to the apoptotic mechanism by inducing apoptosis and gene sequencing [13].

### The origin of eccDNA

EccDNA is widely found in the genomes of organisms and is derived from genomic segments of different chromosomes joining together. Their abundance is associated with the number of genome copies. For example, according to Cohen et al., eccDNA was discovered across the life cycle of Drosophila. These molecules represent approximately 10% of the overall repetitive DNA content and range in size from < 1 kb to > 20 kb. Drosophila eccDNA contains cyclic polymorphs of tandem repeat genes, such as histone genes, rDNA, stellate genes, and suppressors of stellate genes [14]. In regard to human chromosomes, Møller et al. found that chromosomal breakpoints ranging from 0.05 kb to 999.8 kb could form a large number of different eccDNAs. EccDNA is derived from human chromosomes of all known types of genomic structural sequences. In addition, eccDNA is a common mutational element in human bodies [5].

**Fig. 2:**
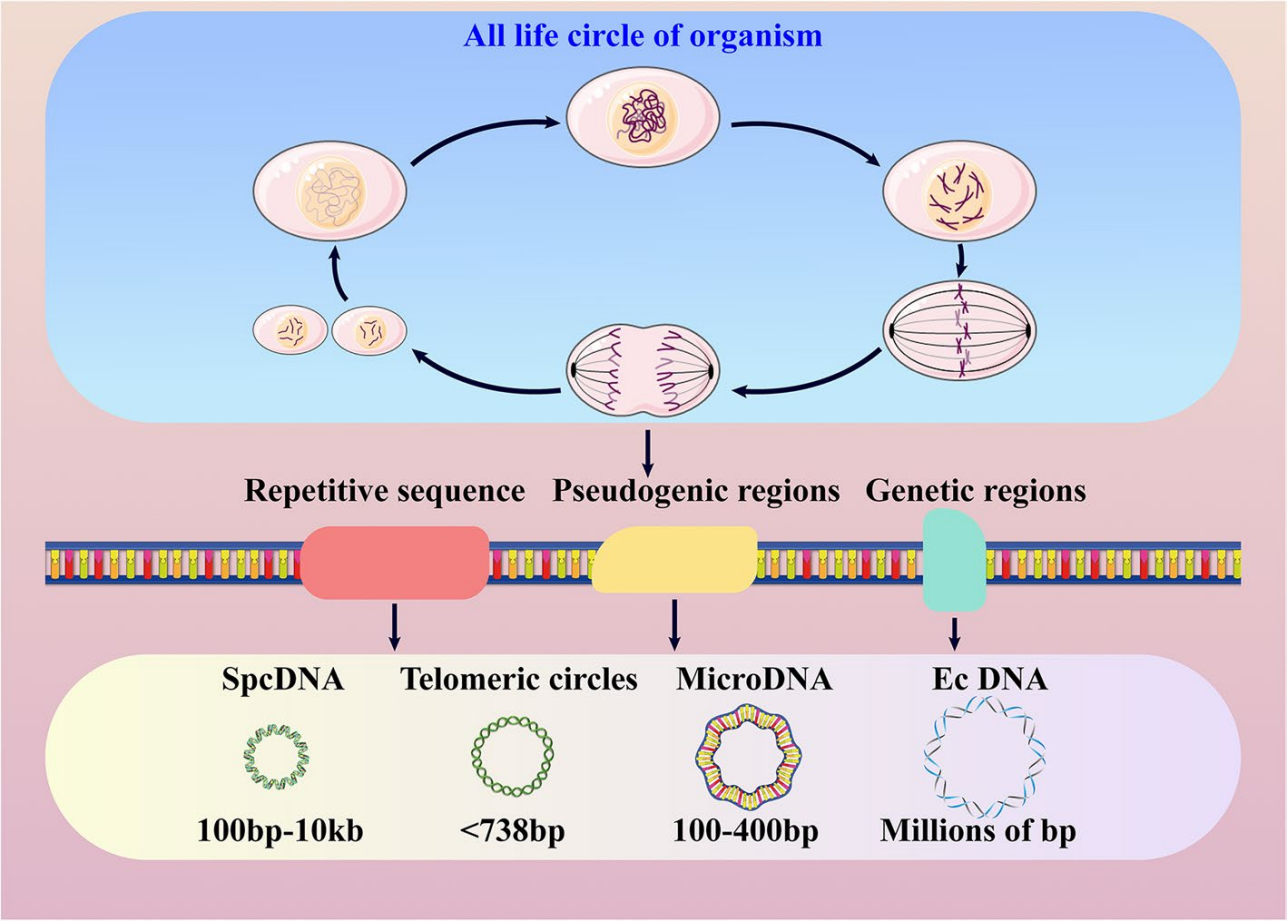
The origin of eccDNA. EccDNA is widely found in all known types of genomic structural sequences and can be created in all life cycles of organisms. Among these sequences, the birth of eccDNA mainly occurs in repetitive sequences, pseudogenic regions and genetic regions. According to the size, eccDNA is divided into four types, namely, spcDNA, telomeric circles, microDNA and ecDNA

Although the magnitude of eccDNA differs greatly, varying from several tens to hundreds or thousands of bases, most of them are smaller than 1000 bp and are derived from repetitive sequences [4, 5]. Up to half of the eccDNA is generated from genetic or pseudogenic regions. According to the various sequence sizes and eccDNA, it can be divided into four categories: small polydispersed DNA (spcDNA) (100 bp-10 kb), telomeric circles (t-circles) (multiples of 738 bp), microDNA (100–400 bp) and ecDNA (millions of bp) [2]. Furthermore, genomic abundance affects the amount of eccDNA produced (Fig. 2). A large amount of eccDNAs is produced by gene-rich chromosomes. Among these regions, titin (TTN), the most frequently transcribed gene to code protein, contributes to the largest number of eccDNAs. The plausible reason for this is that repetitive sequences, gene-rich chromosomes, and tandem paralogous genes have a higher probability of circularizing and shaping eccDNA [5], consistent with the former data from the human germline and yeast [8, 15–17].

### Biogenesis of eccDNA

Although the exact process of eccDNA biogenesis is not yet clear, the biological mechanism of eccDNA generation has been gradually clarified through a series of studies. Some biological processes, including DNA damage repair pathways, chromothripsis, and apoptosis, are related to eccDNA formation [12, 13, 18]. DNA replication and transcription also play important roles in the models mentioned above [19]. In addition, DNA recombination, DNA rearrangement, and other events have also been suggested as potential mechanisms [8, 18–20]. Therefore, more details are needed to thoroughly elucidate the mechanism of eccDNA biogenesis.

#### DNA damage repair pathways

According to the previous section, most of the eccDNA originates from duplicated sequences in the genome. Moreover, most eccDNA contains or is adjacent to short direct repetitive sequences [4]. For instance, among the 30,000 unique eccDNAs, 72.4% (human) and 8.7% (pigeon) were derived from repetitive elements [21]. Moreover, DNA damage factors, such as hydroxyurea (HU), provide evidence for a role of DDR in eccDNA production [22]. The absence of the DNA repair protein called malignant hyperthermia susceptibility 3 (MHS3) caused significantly reduced quantities of circular DNA in human ovarian and prostate cancers, showing that specific proteins in the DNA damage response (DDR), represented by MHS3, are necessary for the formation of eccDNA [23]. Based on this phenomenon, researchers concluded that the production of eccDNA is related to DNA repair pathways, especially homologous recombination (HR) and microhomology-mediated end joining (MMEJ) between two short repeat sequences [24, 25].

A recent study in 2021 showed that different DNA repair pathways after damage have different effects on the amount of microDNA formation [12]. The amount of microDNA increases at double-strand break (DSB) sites and in the S, G2 and M phases of the cell cycle, indicating that DSB induces microDNA production [12]. Cells lacking the canonical nonhomologous end joining (c-NHEJ) pathway produce more microDNA after injury. Thus, microDNA formation may be inhibited by the c-NHEJ pathway. In contrast, the MMEJ pathway may promote microDNA production because the indispensable protein of the MMEJ pathway is able to facilitate the production of microDNA. Additionally, the formation of microDNA is independent of base excision repair, single-strand annealing, and the homologous recombination pathway [12]. Different from microDNA, the production of ecDNA may be induced by the HR pathway and NHEJ pathway. The amount of ecDNA was reduced in MTX-resistant HT-29 colon cancer cells by silencing the breast and ovarian cancer susceptibility protein 1 (BRCA1) gene and the protein kinase, DNA-activated, catalytic subunit (PRKDC) gene, both of which are key players in the HR pathway and the NHEJ pathway, respectively [26, 27].

#### Breakage-fusion-bridge mechanism

Molar et al. suggested that the amount of eccDNA is related to the number of repetitive elements in the genome [21]. Taking *Columba livia* domestica as a model, this experiment explored the amount of eccDNA in a condensed, less repetitive element genome and compared it to that in humans. The results showed that 72.4% of the 30,000 characterized eccDNAs were from humans (expected 52.5%), much higher than those from pigeons [21]. This shows that repetitive sequences are closely related to the production of eccDNA. A large amount of spcDNA was isolated from Chinese hamster ovary cells [28], and the direct repeats contained at both ends of the repetitive sequence of sugarcane cells were cyclized to form eccDNA [29]. The presence of high levels of any direct tandem repeats could confer the ability to convert DNA into circular multimers. Homologous recombination is involved in the formation of eccDNA.

Repeated sequences can be excised by homologous recombination, cyclized and formed into eccDNA [30–32]. Moreover, studies have shown that BFB cycles are strongly associated with chromothripsis [18]. The occurrence of the BFB cycle provides additional instability to the genome. Defective replication of bridge DNA may trigger a much higher chance of secondary DNA damage and even chromothripsis. Chromosomal breakage leads to a complex iterative cycle; thus, division errors in individual cells create multiple hallmark features of cancer [32].

#### Chromothripsis

Severe DNA damage caused by exogenous stimuli leads to chromothripsis, while chromosome breakage leads to the formation of eccDNA. The mechanism of chromothripsis on eccDNA formation is still uncertain. A recent study by Shoshani et al. demonstrates that chromothripsis is one of the main drivers of ecDNA amplification and has a role in promoting gene rearrangement [18]. The mechanism of this process is dependent on poly ADP-ribose polymerases (PARP) and the catalytic subunit of DNA-dependent protein kinase (DNA-PKcs) [18]. Further, treatment of cancer cells with methotrexate may induce chromothripsis and increase DM copy number in a dose-dependent manner [18].

Chromothripsis drives oncogene amplification and then mediates the overexpression of oncogenes, which finally contributes to the development of cancer [11, 33, 34]. Chromothripsis events occurred in 34 of 36 patients with gastric cardia adenocarcinoma (GCA) [35]. The location of this event in the genome was rather heterogeneous in these samples [35]. Whereas some locally amplified oncogene loci, such as the erb-b2 receptor tyrosine kinase 2 (ERBB2) and MYC proto-oncogene (MYC), overlapped significantly with chromothripsis, oncogene amplification was positively associated with chromothripsis events [35]. The occurrence of chromothripsis is over 50% in several cancer types [36], indicating that chromothripsis is linked to tumor genomic instability and DNA damage [37–40].

#### Cell apoptosis

A recent study by Wang et al. suggested that the biogenesis of eccDNA may be positively associated with apoptosis [13]. The production of eccDNA is dependent on the breakage of apoptotic DNA fragments (ADFs), which are then combined with DNA ligase 3 [13]. To determine whether eccDNA production requires ADF, which is induced by caspase-activated DNase (CAD) [41], endonuclease G (EndoG) [42] or DNase γ [43] in a cell-type-specific mode, controlled experiments were performed by purifying eccDNA in UV-treated cells using a novel eccDNA purification method. The removal of ADF may prevent the production of eccDNA, indicating that ADF is a prerequisite for eccDNA production [13].

There are three DNA ligase genes (Lig1, Lig3 and Lig4) in mammals, each with a particular role. The role of these ligases has been well investigated in the CH12F3 mouse B-lymphocyte cell line [44]. Whereas knockdown of Lig1 or Lig4, neither alone nor in combination, had a noticeable effect on the production of eccDNA, knockdown of Lig3 significantly reduced the production of eccDNA [44]. Since double knockdown of Lig1 and Lig3 is lethal to cells [44, 45], it is not clear whether double knockdown can completely eliminate eccDNA production.

### Functions of eccDNA

**Fig. 3:**
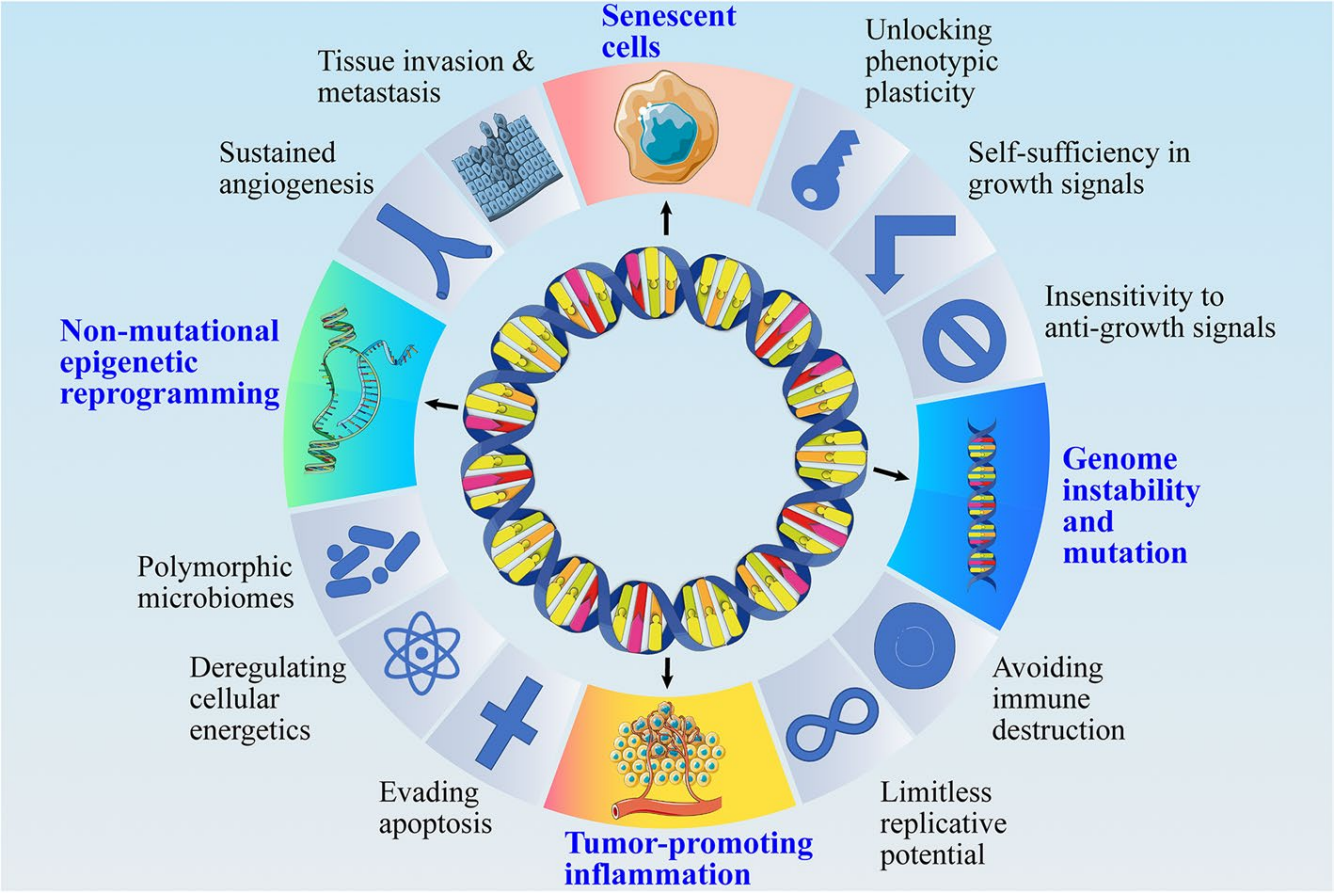
The function of eccDNA. The functions of eccDNA, such as amplification of oncogenes, control of transcription, aggregation in senescent cells and induction of immune responses, are closely related to the hallmarks of cancer. In particular, these 4 of 14 hallmarks include genome instability and mutation, nonmutational epigenetic reprogramming, senescent cells and tumor-promoting inflammation [6, 46, 47]

The characterization of tumors has been a major research topic. From 2000 to 2011, Hanahan and Weinberg successively proposed 10 well-known hallmarks of cancer, namely, self-sufficiency in growth signals, insensitivity to anti-growth signals, evasion of apoptosis, limitless replicative potential, sustained angiogenesis, tissue invasion and metastasis, deregulating cellular energetics, avoiding immune destruction, tumor-promoting inflammation, genome instability and mutation [46, 47]. In 2022, the hallmarks of cancer are updated, which include unlocking phenotypic plasticity, senescent cells, nonmutational epigenetic reprogramming, and polymorphic microbiomes [6]. In this section, we explore and classify the functions and mechanisms of eccDNA based on the latest tumor hallmarks. The functions of eccDNA are closely related to several cancer hallmarks, including tumor cell instability and mutation, heterogeneity, cellular senescence, and tumor proinflammatory response (Fig. 3).

#### The related mechanisms of eccDNA

**Fig. 4:**
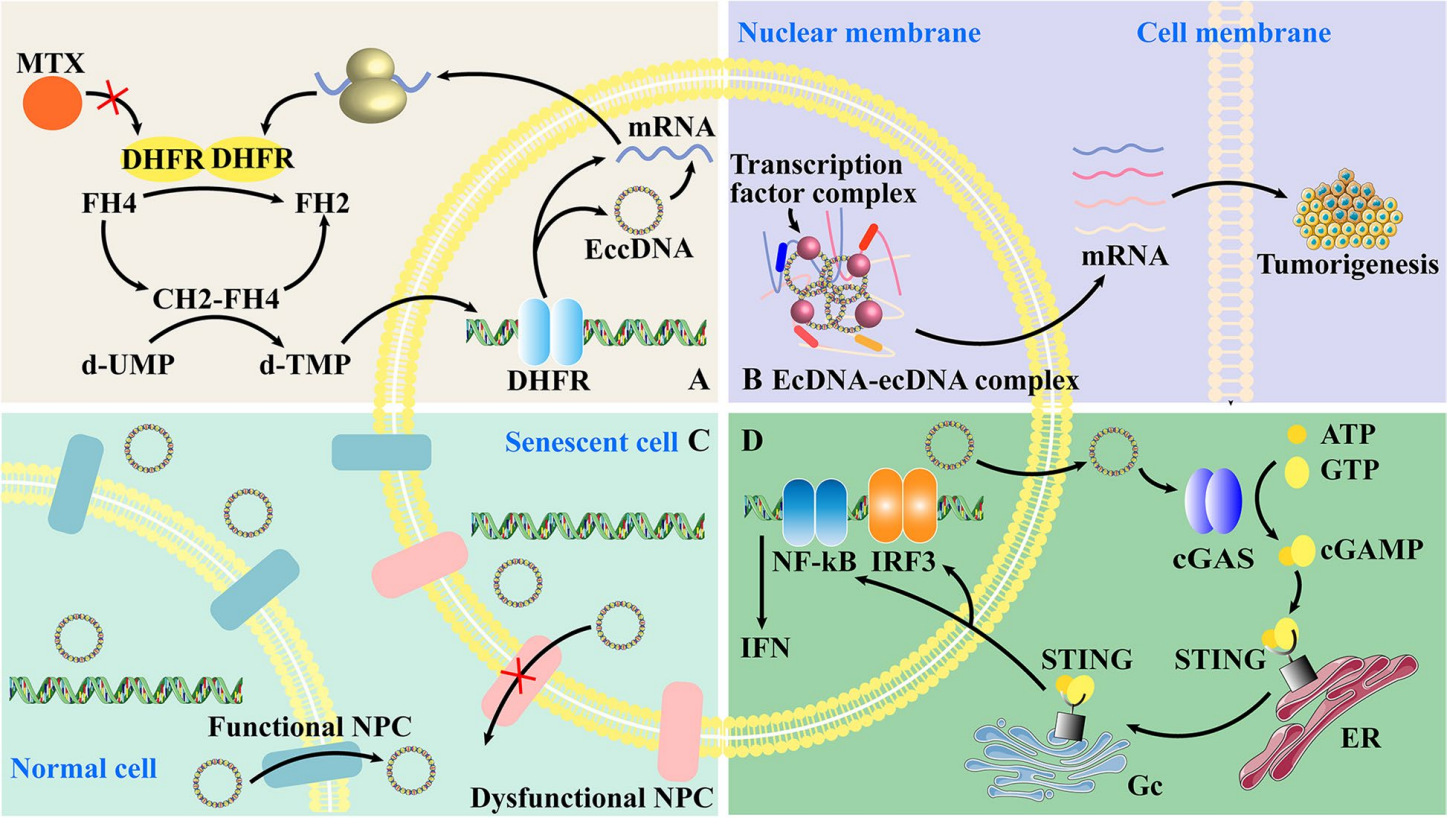
The mechanism of eccDNA. A EccDNA amplifies the DHFR gene on the chromosome. Overexpression of the DHFR gene leads to a large amount of DHFR in cells; thus, methotrexate (MTX) cannot effectively prevent DHFR from participating in the interchange of FH2 and FH4, which convert d-UMP to d-TMP. The d-TMP is helpful for gene replication, leading to instability and mutation of the tumor. **B** Transcriptionally active ecDNAs are polymerized by the transcriptional complex and become enhancers that regulate transcription. This enhancer functions by binding to the relevant chromosomal genes, thereby activating the expression of genes associated with tumorigenesis. **C** In senescent cells, functional NPCs on the nuclear membrane tend to become dysfunctional, which means that cells are not capable of excluding eccDNA from the extranucleus. Gathering in the nucleus can largely be defined as a symbol of senescent cells. **D** EccDNA is manufactured, leaves the nucleus, and activates the cGAS protein in tumors. ATP and GTP thus form into cGAMP, combing with STING protein on the endoplasmic reticulum (ER), combining STING protein to change its position to the Golgi complex (GC). This whole process is named the cGAS-STING pathway. The chromosomal genes NK-κB and IRF3 are stimulated by this pathway, processing IFN and immune-related cytokines and then inducing the immune response in the body

Before specifically analyzing the effects of eccDNA on tumor hallmarks, we first briefly summarize the mechanisms associated with eccDNA (Fig. 4). The major mechanism of eccDNA is gene amplification [35]. By amplifying oncogenes, eccDNA helps tumor cells create numerous products to grow, mutate and invade. For example, the amplification of the DHFR gene gives tumor resistance to MTX by increasing the amount of DHFR produced [18, 34]. Moreover, eccDNA induces gene rearrangement in somatic cells, resulting in gene instability and characteristic changes [48]. The unequal division of eccDNA also contributes to the heterogeneity of tumor genes, adding challenges to clinical drug development.

EccDNA is able to gather together, becoming a hub to enhance transcription. Tumorigenesis can be activated by ecDNA hubs and inhibited by the destruction of these hubs [49]. The existence of hubs also increases chromatin accessibility and is a perfect target for clinical detection because ecDNA hubs are not as intense as chromosomes [50]. Moreover, ecDNAs have extensive ecDNA-ecDNA interactions and ecDNA-chromosome interactions. Thus, ecDNA-ecDNA can act as a mobile transcriptional enhancer to promote tumor progression [51].

EccDNA is also related to cellular senescence. It is obvious that eccDNA accumulates largely in aging cells, not just because of the years of growth. For example, aging yeast will accumulate circular DNA encoded by high-copy proteins obtained through random and environmentally stimulated recombinant processes [52]. The mechanism that excludes eccDNA from the nucleus gradually malfunctions with cellular senescence, leading to a massive accumulation of eccDNA in the nucleus [53, 54].

In a recent study, a relationship between eccDNA and apoptosis was also found [13]. Moreover, eccDNA is responsible for activating the immune system of the human body. The occurrence of eccDNA impacts the cyclic GMP-AMP synthase (cGAS) pathway and stimulator of interferon genes (STING) pathway, leading to the overexpression of the interferon (IFN) gene [13]. In addition, when eccDNA is released into circulation, this kind of circular cell-free DNA contains enough information. It may mediate the communication between cells and signal transduction, as exosomes do [55].

In general, various mechanisms endow eccDNA with abundant functions related to tumors. It is necessary to explore the potential associations between eccDNA and tumor hallmarks.

#### eccDNA is involved in the formation of oncogene instability and mutations

The acquisition of multiple hallmarks of tumors depends to a considerable extent on the instability and mutation of the tumor cell genome. It confers a differentiation advantage to tumor cells, allowing them to remain dominant in the local microenvironment. In addition, the instability makes tumors resistant to chemotherapeutic agents, making targeted therapy and drug development for tumors difficult. A combination of factors leads to the creation of tumor genomic instability. DNA methylation and histone modifications, for example, can be acquired through epigenetic mechanisms, while certain mechanisms, by affecting the regulation of gene expression without relying on mutational triggers, can likewise induce oncogene amplification and advance cancer.

Focal amplification of genes has a critical role in tumor cell growth, driving tumor cell evolution, and is associated with poor prognosis of tumors. A series of studies have shown that eccDNA can advance cancer by mediating gene amplification. In yeast, eccDNA is involved in gene amplification, thereby promoting yeast cells to develop adaptations to nutrient-limited environments [8, 56]. Moreover, in plants, the presence of eccDNA enhances resistance to herbicides [57]. EccDNA may provide an efficient pathway for gene amplification by evolving to cause genomic changes in cancer cells. Taking some GCA patients as an example, ERBB2/EGFR is present as circular DNA in cancer cells and forms focal amplification. In addition, local amplification was observed in 80% of patients with cancer cells, and coamplification of two or more oncogenes occurred in more than half of them. In these 10 tissues of GCA patients, cyclic DNA was present [35]. Moreover, noncoding enhancer elements are often amplified with oncogenes on eccDNA, contributing to the overexpression of oncogenes. It thus provides an advantage for tumor growth and drug resistance; for example, the overamplification of DHFR leads to the development of tumor resistance to methotrexate [11].

EccDNA also regulates oncogene expression by participating in transcription [4, 49, 51]. First, when glyphosate-resistant plants containing replicons were exposed to glyphosate, 49 of 59 genes encoded by eccDNA replicons were transcriptionally active [57]. EccDNA may act as a transcriptional element, such as an enhancer or promoter, to promote gene expression, and the regulation of RNA expression by sponge transcription factors is also a theoretical function of eccDNA, which has yet to be further verified by researchers [49, 58]. Second, eccDNA promotes oncogene overexpression by activating cis-regulatory elements on the same chromosome [50, 59, 60]. The distribution of eccDNA in cells was visualized by fluorescence in situ hybridization (FISH), and eccDNA was found to be highly aggregated, called eccDNA hubs. EccDNA hubs are major sites of oncogene transcription: BRD4 connects ecDNA hubs to initiate oncogene transcription, while disruption of ecDNA hubs triggers repression of transcription and cell death [49]. In addition, ecDNA has extensive ecDNA-ecDNA interactions and ecDNA-chromosome interactions that have important transcriptional regulatory functions. EcDNA can act as a mobile enhancer that regulates the activation of transcription [51]. Moreover, eccDNA from gene-rich chromosomal regions may affect somatic cell genotypes by altering gene copy number [5]. Compared to chromosomal DNA, eccDNA may lead to an increase in the copy number of oncogenes, resulting in products with high expression of oncogenes. Associated studies have shown that oncogenes encoded on ecDNA are among the most highly expressed genes in the tumor transcriptome. When ecDNA is spatially bundled with other ecDNA aggregates, this bundling, with one ecDNA as the core and other ecDNAs as auxiliaries, makes the transcription of proto-oncogenes much more likely [49]. However, although ecDNA is packaged as chromatin with complete structural domains, it lacks the higher-order densities typical of chromosomes, which significantly enhances chromatin accessibility [50].

EccDNA were significantly enriched in gene regions, especially in MYCN-amplified neuroblastoma. A small subgroup of genes that were completely cyclized, such as NTF3, were significantly increased in expression by RNA sequencing and amplified as eccDNA. Most of the **i**nterchromosomal and intrachromosomal rearrangements detected in the neuroblastoma genome were consistent with extrachromosomal looping regions, demonstrating that eccDNA may mediate genomic rearrangements [48]. Such genomic rearrangements may promote aberrant expression of tumor suppressors and proto-oncogenes.

#### eccDNA is associated with the heterogeneity of tumor cells

In a recent study, ecDNA hubs were shown to span more than 1000 nm and contain trans-regulatory elements located on different ecDNA molecules. This finding has profound implications for how ecDNA undergoes selection and how oncogene regulation of ecDNA contributes to transcription. First, trans-activation between ecDNA suggests that coselection of oncogene enhancers may occur on individual ecDNA and on all ecDNA within the cell. Thus, a single ecDNA molecule may not need to contain all the necessary regulatory elements. Different enhancers between cells may lead to tumor heterogeneity and provide additional evolutionary directions for tumor adaptation to the environment [49].

The extensive, random distribution of eccDNA across the genome results in the formation of eccDNA that varies across changes in DNA, creating a diversity of eccDNA [34]. In addition, due to the lack of mitotic granules, eccDNA often segregates randomly in mitosis and replicates independently from mitosis of cells, leading to significant heterogeneity in tumor cells [61, 62]. It is worth mentioning that the function of eccDNA to accelerate oncogene copies also advances the heterogeneity of tumor cells. Patients with intracellular eccDNA have a worse prognosis than those with noncyclic amplification [35, 48].

#### eccDNA is associated with cellular senescence

Cellular senescence has long been recognized as a defense mechanism against tumors. Cancer cells are induced to senescence to prevent their proliferation, and senescence has a protective role in limiting malignant progression [63]. However, recent studies have shown that the role of senescent cells may be quite different from what was expected. In some cases, senescent cells stimulate the development and progression of tumors. For example, depletion of senescent mouse cells reduces the incidence of tumorigenesis and associated death [6].

In senescent cells, eccDNA accumulates heavily due to the weakening of the rejection mechanism. The accumulation of eccDNA-containing ribosomal RNA genes over time promotes the aging of yeast cells [52]. EccDNA is formed mainly from DNA damage repair. They then follow nuclear actin to the nuclear pore complexes (NPCs) and are eventually excluded from the nucleus by functional NPCs in cells with normal functions [53, 54]. In senescent cells, various cancers and several other age-related diseases (ARDs) (cardiac diseases, premature aging, neurodegenerative diseases and myopathies, for example), however, dysfunctional NPCs, nuclear actin rods and aberrant NPC components have been demonstrated to be augmented [64, 65]. Thus, in senescence and in the ARDs mentioned above, the accumulation of eccDNA in the nucleus is mainly due to an increase in dysfunctional NPCs, which reduces the rejection of nuclear eccDNA.

#### eccDNA participates in the tumor proinflammatory response

Almost every tumor lesion contains the involvement of immune cells, ranging from tiny infiltrates visible only at the cellular level to significant and severe inflammation. Tumor-associated inflammation was once thought to be the body’s response to try to eliminate the tumor, but as experiments progress, there is increasing evidence that immune cells, especially the innate immune system, have an important role in promoting tumor progression [66, 67].

EccDNA is considered to induce the production of innate immunity. Cells respond to naked DNA in the cytoplasm by activating the cGAS pathway, which ultimately expresses interferon and stimulates the immune system, one of the pathways of the intrinsic immune response. Researchers hypothesize that eccDNA is excluded into the cytoplasm during mitosis, where it is either degraded by enzymes such as three prime repair exonuclease 1 (TRX1) or activated by the cGAS pathway [68, 69]. In general, it acts as an endogenous. Compared to linear DNA, circular DNA dramatically improved gene expression, including cytokines and chemokines. Nine of the top 20 most upregulated genes belonged to the family of type I interferons. In addition, they are richly associated with both immune responses and relevant signaling pathways, reflecting that eccDNA is a natural stimulator of immune responses [13].

Extracellular vesicles (EVs) include exosomes apoptotic bodies and microvesicles. They are released by cells, including cancer cells, into the surrounding biological fluid or circulation. These exosomes contain tumor-derived materials such as DNA, RNA, proteins, lipids, glycan structures and metabolites. Cancer cells often use them as biological messengers for communication. EccDNA enhances the expression of relevant oncogenes through amplification, and the upregulated products, such as EGFR and IFN, are delivered by EVs to complete tumor cell communication, migration, and the body’s immune response [70]. Furthermore, cell-free eccDNA (30–60% > 250 bases) is significantly longer than cell-free circulating linear DNA (∼150 bases); thus, eccDNA is a previously unexplored nucleic acid pool [55]. eccDNA may be able to complement miRNA and linear DNA for diagnostic and intercellular communication.

### Possible applications of eccDNA in cancer diagnosis and treatment

The function of eccDNA in relation to tumors provides an alternative approach to the clinical diagnosis and treatment of cancer. Following the progression of cancer, we analyze the significance of eccDNA for clinical research in oncology from three stages. First, in the diagnosis and detection of early-stage tumors, eccDNA could serve as a reliable biologic signature and an effective biomarker. Second, in the clinical treatment of tumors, an eccDNA-targeted tumor therapy deresistance strategy is expected to improve the efficacy of eccDNA-induced tumor therapy resistance. Finally, in the prognostic stage of tumors, eccDNA can be used as an effective means to predict patient prognosis.

#### Application potential of eccDNA in the early diagnosis of cancer

Today, we still face many difficulties in the treatment and monitoring of cancer. Research on eccDNA may provide us with new ideas and directions. Crosby et al. summarized the challenges of early cancer detection and monitoring, including the need for researchers to understand the biology of early cancers and to continuously search for and validate effective biomarkers [71]. The absence of a clear precancerous lesion in some cancers and the fact that not all precancerous lesions eventually develop into cancer mean that we have not yet been able to identify a sufficiently accurate biologic signature [72]. Researchers have identified the immune system as a key regulator and assessor of early cancer development [73], and immune cells and their participating molecules contribute to the early detection of cancer. Combined with the role of eccDNA in the induction of the body’s immune response this role may become a common biological feature of different types of cancer [13],. If early-stage cancers are effectively detected, diagnosed and treated, the survival rate of patients will be significantly improved.

Although several biomarkers for early cancer surveillance are available, few have been validated and applied in clinical trials. For example, mutations in the KRAS proto-oncogene are associated with disease progression in colon cancer but are unable to accurately diagnose the development of pancreatic cancer [74]. Therefore, ideal biomarkers need to be actionable and have sufficient predictive value for prognosis. In recent years, liquid biopsy has become an important modality for the early diagnosis of tumors. By analyzing and testing body effluent (such as blood or urine), liquid biopsies allow to monitor the evolution of cancer in real time [75, 76]. The rapid development of next-generation sequencing technologies and advances in the detection of circulating tumor DNA have brought liquid biopsy into clinical practice [77]. The clinical detection of tumors has entered a new phase of noninvasive, real-time testing. Liquid biopsies are performed on circular tumor cells and cell-free DNA (cfDNA) in the blood. When the blood, brain, muscle and other tissues of cancer patients release eccDNA into the circulation [5, 19], the presence of eccDNA in the form of cfDNA undoubtedly provides a large number of targets for liquid biopsies [55]. EccDNA is produced in large quantities during tumorigenesis, accompanied by the generation of a series of epigenetic modifications to DNA. The development of multiple biomarkers, resulting in a multimodal assay, could achieve higher sensitivity and specificity.

#### Application potential of eccDNA in the treatment of cancer

Medium-term cancer treatment has always been plagued by tumor drug resistance, rapid adaptation to new environments, etc., eccDNA amplifies genes, regulates transcription, and divides unequally, providing a new perspective on the characteristics of tumor cells. The amplification of genes on eccDNA and the regulation of transcription are closely related to the drug resistance of tumor cells. For example, amplification of DHFR by eccDNA causes its overexpression and leads to tumor cell resistance to methotrexate. However, the amount of eccDNA and the expression level of DHFR were significantly decreased in successive passages in drug-free medium [11]. In addition, the amount of eccDNA was positively correlated with the concentration of methotrexate at different concentrations of methotrexate [36]. The effect of external drugs stimulated an increase in eccDNA, which led to the amplification of drug resistance genes in tumors and the eventual acquisition of drug resistance. Blocking the production of eccDNA can lead to a significant decrease in the expression of the related drug resistance genes. There is other similar evidence. *Amaranthus palmeri*, a widespread glyphosate-resistant (GR) weed in the US, gains resistance by overexpressing 5-enolpyruvyl-shikimate-3-phosphate synthase (EPSPS). For GR, genetic analysis revealed a 5- to 160-fold increase in the copy number of its EPSPS gene compared to the glyphosate-sensitive group. The eccDNA containing the EPSPS gene was found to be 400 kb in size [78, 79]. In patients with hypopharyngeal squamous cell carcinoma, a portion of the highly expressed genes involved in cisplatin (DDP) resistance are transcribed entirely or partially by eccDNA. EccDNA amplifies the gene encoding RAB3B, which promotes DDP resistance in hypopharyngeal squamous carcinoma by inducing autophagy [80]. This is the predominant mechanism by which DDP resistance arises.

Using this conclusion, we can control the drug resistance of tumor cells by blocking the production of eccDNA. Studying the DNA damage repair pathway after DSB, MMEJ, chromothripsis and other pathways to produce eccDNA may lead to effective biological targets. A decrease in eccDNA will result in a reduction in resistance gene expression, causing tumors to lack drug resistance when treated. Moreover, the protein produced by eccDNA can also become an effective therapeutic target in clinical treatment.

#### Potential application of eccDNA in prognostic warning of malignant tumors

EccDNA is also highly correlated with poor prognosis of cancer. EccDNA enables amplification of oncogenes, and different amplification sites have different prognostic effects on cancer. For example, a high copy number of EGFR and MYC in pancreatic cancer patients predicts a shorter survival time, while in contrast, a high copy number of CCNE1, ERBB2, etc., predicts a longer survival time [35].

Through computational evaluation of the whole genome sequencing (WGS) database of 3212 patients with cancer, Kim et al. found that ecDNA amplification occurred more often in the majority of cancer types than in blood or normal tissue [81]. Oncogenes appear to be highly abundant on amplified ecDNA. EcDNA is more frequent in high frequency invasive histologic cancers such as glioblastoma, sarcoma and esophageal cancer. Upon investigation, patients with tumors containing amplified ecDNA had worse 5-year survival outcomes, showing that the existence of ecDNA is correlated with the aggressiveness of tumors [81]. In human cancers, researchers concluded that ecDNA amplification has a negative effect on patient prognosis, an effect that is independent of the cancer spectrum [81]. This finding is inconsistent with the amplification of ERBB2 in GCA mentioned above because patient survival is due to the diversity in the structure and behavior of eccDNA across cancers. In addition, both nuclear and mitochondrial-derived ecDNA can be used as prognostic markers for ovarian cancer. Compared to the quantification of tumor-derived ecDNA, no understanding of tumor-specific mutations is essential for analyzing these kinds of ecDNA [82]. Large amounts of eccDNA have been detected in patients with rheumatoid arthritis and sepsis [83, 84]. After using deoxyribonuclease to remove ecDNA from extracellular fluid, the patients’ symptoms were relieved to some extent. Although there are many sources of eccDNA and the structure and behavior of eccDNA vary in different cancers or diseases, there is no doubt that eccDNA has a great impact on the prognosis of patients.

Advanced technologies make it feasible to accurately track the large amounts of eccDNA produced in cancer. The combined use of WGS and whole-exome sequencing has been validated to detect and count the presence of eccDNA with relative accuracy. Moreover, the use of an amplicon reconstruction instrument allows the identification of focal amplification and ecDNA from WGS data. Amplicon Architect is capable of selecting eccDNA according to its biological characteristics. Further studies on eccDNA-amplified oncogenes can help to predict cancer prognosis more reasonably. Investigators can further clarify the prognostic impact of eccDNA on different cancers by exploring the behavioral specificity of eccDNA in different tumors. WGS combined with Amplicon Architect can accurately select eccDNA from chromosomal DNA, and Circle-seq will examine the mistakes from WGS. Using eccDNA as an effective predictor of prognosis is quite feasible.

### Summary and perspectives

Thanks to the development of technology and the evolution of research methods, the complex and important functions of eccDNA have been uncovered and have attracted a great deal of attention from researchers. Damage repair pathways, the BFB cycle and chromothripsis are the biogenesis mechanisms of eccDNA, which have been proven by numerous studies. Moreover, in the latest study, the specific mechanism by which apoptosis produces eccDNA has been investigated and confirmed [13], and we are one step closer to our target.

In the field of oncology, the exploration of the function of eccDNA is even more important. Thanks to the advancement of detection tools, we have explored whether eccDNA can affect tumor instability, drug resistance, and heterogeneity and cause an immune inflammatory response and cellular senescence through mechanisms such as gene amplification, transcriptional regulation, rearrangement of oncogenes, unequal division, massive accumulation in senescent cells, signal transduction between tumor cells and induction of the immune response, ultimately promoting its malignant process. Nevertheless, we still need to address the shortcomings and unsolved mysteries in eccDNA research. The specific mechanisms of eccDNA biogenesis are still not precisely studied, and the sequencing methods of eccDNA need to be updated and improved to identify eccDNA from chromosomal DNA. The mechanisms of how eccDNA is generated, how it is recombined, and how to maintain the balance of quantity need to be further explored. Although the biogenesis of eccDNA has been studied in tumors and a new study has discovered that there is a combination between cell apoptosis and the production of eccDNA, it is still unclear whether pyroptosis, ferroptosis, copper death, and necroptosis generate new functional eccDNA. This may bring us a new perspective for its role in tumor development.

In addition, the specific functions of different types of eccDNA lack detailed differentiation and proper summary; the impact of eccDNA on cancer prognosis needs to be further investigated. Liquid biopsy is now an effective tool for cancer surveillance, but the sensitivity to eccDNA, the exclusion of artifacts upon eccDNA detection, and the lack of specific targeted solutions still need to be solved. It requires researchers to improve their research methods and ideas to solve the unknown puzzles. Currently, the development and utilization of eccDNA in the field of cancer is still in its early stages. We firmly believe that the application of eccDNA may be a solid supplement to the early screening, real-time monitoring, prognosis evaluation and treatment of cancer and should encourage further studies in this field.

## Data Availability

Not applicable.

## Abbreviations

EccDNA: Extrachromosomal circular DNA
BFB: Breakage-fusion-bridge
DMs: Double minutes
SpcDNA: Small poly-dispersed DNA
T-circles: Telomeric circles
EcDNA: Extrachromosomal DNA
DHFR: Dihydrofolate reductase
EGFR: Epidermal growth factor receptor
TTN: Titin
HU: Hydroxyurea
MHS3: Malignant hyperthermia susceptibility 3
DDR: DNA damage response
HR: Homologous recombination
MMEJ: Microhomology-mediated end joining
DSB: Double-strand break
c-NHEJ: Canonical nonhomologous end joining
BRCA1: Breast and ovarian cancer susceptibility protein 1
PRKDC: Protein kinase, DNA-activated, catalytic subunit
PARP: Poly ADP-ribose polymerases
DNA-PKcs: DNA-dependent protein kinase
GCA: Gastric cardia adenocarcinoma
ERBB2: Erb-b2 receptor tyrosine kinase 2
MYC: Myc proto-oncogene
ADFs: Apoptotic DNA fragments
CAD: Caspase-activated DNase
EndoG: Endonuclease G
Lig: Ligase
cGAS: Cyclic GMP-AMP synthase
STING: Stimulator of interferon genes
IFN: Interferon
FISH: Fluorescence in situ hybridization
NPC: Nuclear pore complex
ARDs: Age-related diseases
TRX1: Three prime repair exonuclease 1
EVs: Extracellular vesicles
CfDNA: Cell-free DNA
GR: Glyphosate-resistant
EPSPS: 5-enolpyruvyl-shikimate-3-phosphate synthase
DDP: Cisplatin
WGS: Whole genome sequencing
MTX: Methotrexate
ER: Endoplasmic reticulum
GC: Golgi complex
